# Metabolic Activity of Invasive Apple Snails Negatively Affects the Survival of Native Benthic Snail in Mangrove

**DOI:** 10.3390/biology14020141

**Published:** 2025-01-29

**Authors:** Jinling Liu, Caiying Zhang, Huixiu Yu, Zixin Fu, Huizhen Xie, Yiming Wang, Benliang Zhao, Qing Li, Kailin Kuang, Huanting Lin

**Affiliations:** 1 College of Biology and Food Engineering, Guangdong University of Education, Guangzhou 510303, China; liujinling09@163.com (J.L.);; 2 College of Natural Resources and Environment, South China Agricultural University, Guangzhou 510642, China

**Keywords:** *Pomacea canaliculata*, *Neritina pulligera*, mangrove forest, interspecific competition, water quality

## Abstract

The golden apple snail (GAS, *Pomacea canaliculata*) has invaded mangrove forests. The effect of water contaminated by metabolic activity of GAS feeding on *Acanthus ilicifolius* (T1), *Sonneratia apetala* (T2), and without food (CK) on the native mangrove black helmet snail (BHS, *Neritina pulligera*) was investigated under salinity conditions. The GAS deteriorated saline water quality (2.5‰). DO contents in T1 and T2 approached zero at 9 d. Compared to CK, the contents of COD, total N, NH_4_^+^, NO_3_^−^, and total P of the contaminated water in T1 increased by 297%, 205%, 262%, 210%, and 518% after 9 d, while these indicators in T2 increased by 74%, 31%, 57%, 326%, and 154%, respectively. The LC_50_ of the contaminated water in T1 against the BHS reached 22.72%. The weight of the BHS exposed to the 100% contaminated water in T1 and T2 significantly decreased after exposure. The content of GPT of the BHS exposed to the 100%-contaminated water in T1 and T2 increased by 55% and 26%, while the MDA content increased by 38% and 34%. The 100%-contaminated water in T1 led to cell degeneration and incomplete structure in the hepatopancreas tissue of the BHS. The GAS feeding on holly mangroves can compete against native mangrove snails through water deterioration.

## Simple Summary

The golden apple snail (GAS) is an invasive gastropod worldwide. The interspecific competition between GAS and native snails in mangrove habitats remains unclear. GAS activity significantly alters the water quality in local habitats, possibly affecting the native benthic snail. We investigated the possible disturbance of GAS metabolic activity on native benthic black helmet snails by measuring the water quality, mortality, growth traits, and hepatopancreas tissue after feeding two local mangrove leaves based on their palatability for GAS. GAS activity significantly deteriorated the water quality in 2.5‰ saline water. GAS feeding on two mangroves decreased the values of pH and DO while increasing the contents of COD, total N, NH_4_^+^, NO_3_^−^, and total P. The mortality, weight and physiological traits, including GPT, GOT, MDA, and protein, were significantly affected by different dilution ratios (0–100%). Snails exposed to the contaminated water in T1 showed a maximum mortality. Furthermore, the structure of hepatopancreas tissue in the BHS was significantly damaged by the contaminated water from the GAS feeding on holly mangrove. The GAS can compete against the native mangrove snails through water quality deterioration related to the feeding behaviors of different mangrove species.

## 1. Introduction

The golden apple snail (GAS, *Pomacea canaliculata* Lamarck, 1822) is an aquatic gastropod native to the tropical and subtropical regions of South America. The GAS has become an invasive species in aquatic ecosystems worldwide, including lakes, canals, ponds, and paddy fields. The GAS has become one of the 100 malignant invasive species that endanger biodiversity, agricultural production, and human health in the world [1]. As a benthic invasive snail, the GAS shows strong adaptability, diverse diets, large egg production, high reproductive rate, and rapid growth in invaded habitats. Consequently, GAS invasion leads to inevitable competition with indigenous snails with similar niches for resources and space [2]. In most cases, the GAS eventually succeeds in such competition and occupies a dominant position in new habitats due to its strong ability in reproduction, environmental adaptability, and feeding behaviors. Furthermore, the GAS can compete against local benthic snails in an indirect way. Usually, the GAS feeds heavily on the plant tissues in aquatic environments, and a large amount of excrement produced during metabolism has led to the deterioration of water quality, especially the fluctuation of pH, dissolved oxygen, and ammonium [3]. Under such a scenario, water can be contaminated by the metabolic activity of the GAS in a small habitat [4], and populations of local species in the invaded habitat experience fluctuations, replacement, and extinction. The loss of species diversity caused by the GAS weakens the resistance of the invaded ecosystem to external disturbances [5].

Mangroves are shrubs and trees with prop roots and pneumatophores that grow in coastal habitats in subtropical and tropical areas. They have significant ecological value such as windbreaks, embankment stabilization, purifying the marine environment, sustaining diverse habitats for marine organisms, and providing food for humans [6]. However, due to the strong disturbance from human activities in the past two decades, mangrove forests on coastlines have suffered biological invasion. Studies have shown that mangroves have been invaded by many exotic animals, such as teak defoliator moths (*Hyblaea puera*), European shore crabs (*Carcinus maenas*), portunid crabs (*Charyhdis hellerii*), green mussels (*Perna viridis*), Cuban tree frogs (*Osteopilus septentrionalis*), prawns (*Macrobrachium rosenbergii*), black mangrove cichlids (*Tilapia mariae*), green iguanas (*Iguana iguana*), lionfishes (*Pterois volitans*/*miles*), and the pacific oyster (*Crassostrea gigas*) [7,8,9,10,11,12,13,14,15,16].

The GAS can tolerate salt stress to some extent. A previous study found that the GAS survived in brackish water up to 5 ppt, although it is a freshwater snail [17]. Recent studies reported that the GAS has invaded the mangrove forest and established a self-sustaining population [18,19]. Considering the negative effect of the GAS on freshwater wetlands, the competition between GAS and local benthic snails may affect the structure of the food web in mangrove forests, possibly threatening the integrity and function of mangrove ecosystems. There is a difference in the tolerance to the change in water quality between the GAS and the local snail. Mangroves have a greater variety of characteristic micro-topographies except for salinity caused by their coastal environment. Water quality deterioration by GAS metabolic activity is possibly involved in an indirect competition process between the GAS and local snails [20], which may play an essential regulatory role in the invasion, thereby providing the GAS with sufficient living space and food sources in mangrove wetlands.

Up to now, there is little research on the mechanism of GAS invasion in mangrove forests. We do not know whether indirect interspecific competition between the GAS and native snails affects the invasion process of the GAS in these habitats. As a native benthic gastropod, the black helmet snail (BHS, *Neritina pulligera* L.) can live in mangrove forests, showing a sensitive response to habitat changes [21]. Considering black helmet snails existed extensively in mangrove forests located in Guangzhou, which was invaded by the GAS, this study aims to know whether water deterioration from the metabolic activity of the GAS affects the native snail. Here, we hypothesized that 1. the metabolic activity of the GAS living in brackish habitats negatively affects the water quality, aiming to quantify the changes in the water indicators under two feeding conditions; 2. water contaminated from GAS metabolic activity was toxic to native black helmet snails under a brackish environment, trying to clarify the survival status of native snail exposure to two types of contaminated water; and 3. the physiological and structural characteristics of black helmet snails significantly changed after exposure to water contaminated by the metabolic activity of the GAS, intending to understand the toxic process of the contaminated water by the GAS. This study helps to understand the GAS invasion process in mangroves and may provide insights into the risk assessment of GAS invasion in mangrove wetlands.

## 2. Materials and Methods

### 2.1. Materials

The mangrove GAS (golden apple snail, *Pomacea canaliculata* Lamarck 1822) and BHS (black helmet snail, *Neritina pulligera* L.) were used to analyze the interspecific competition. GAS is the offspring of salt–tolerant apple snails cultivated in the laboratory for 1 year. BHS were cultivated for 3 months before use in the experiment. The leaves of holly mangrove (*Acanthus ilicifolius* L.) and mangrove apple (*Sonneratia apetala* Buch.) were used as food for GAS due to their palatability [19]. All of these experimental materials were collected in the mangrove wetland of Nansha District, Guangzhou, China (22.620° N, 113.660° E). It is the largest coastal mangrove wetland in Guangzhou, belonging to a typical southern subtropical monsoon maritime climate, with an average annual temperature of 23.4 °C, an average relative humidity of 68%, and an annual precipitation of 1748.9 mm [22].

### 2.2. Collection of the Contaminated Water by GAS

GAS and BHS were separately placed in 2.5‰ saline water for 3 days before the experiment. Twenty GAS individuals (Height 3.5 ± 0.2 cm) were placed in clean plastic boxes (top diameter 40 cm × bottom diameter 28 cm × height 18 cm) filled with 10 L of 2.5‰ saline solution. In order to avoid water contamination from the residual food, decayed mangrove leaves (3 g) of *Acanthus ilicifolius* or *Sonneratia apetala* were fed every day based on the reported feeding traits of GAS [18]. All the decayed mangrove leaves supplied in the experiment were consumed by GAS before the collection of experimental water. Two treatments, including *Acanthus ilicifolius* (T1) and *Sonneratia apetala* (T2), were designed and GAS without feeding any food was used as the control (CK). Two treatments and CK were performed three times. Food residues in the box were promptly cleaned up every day. Contents of temperature, dissolved oxygen (DO), chemical oxygen demand (COD), pH, ammonium (NH_4_^+^), nitrate (NO_3_^−^), total phosphorus (TP), and total nitrogen (TN) of metabolic solution in T1, T2, and CK on the 1, 3, 5, 7, and 9 days were measured accordingly (Table 1).

### 2.3. Effect of Golden Apple Snail Metabolites on Black Helmet Snails

The water contaminated by metabolic activity of GAS in T1 (*Acanthus ilicifolius*), T2 (*Sonneratia apetala*), and CK (without feeding) was collected and stored at −20 °C. Based on a 100% concentration of the solution, gradients of 25%, 50%, and 75% were prepared using 2.5‰ saline solution. The 2.5‰ saline solution was used as a 0% gradient. The contaminated water in T1, T2, and CK of five gradients (0, 25%, 50%, 75%, and 100%) was placed in clean plastic boxes (top diameter 40 cm × bottom diameter 28 cm × height 18 cm) for toxicity test. Ten individual BHS were placed in each box. A gradient of 0% was used as the control. Four gradients of treatments and the control were repeated three times. BHS was fed with 1‰ (*v*:*v*) commercial microalgae (*Chlorella vulgaris*) (Bainuo, Yancheng, China) every three days according to the product instruction. The BHS weight was measured using a balance (0.0001 g, DL91150, Deli Group Co., Ltd., Foshan, China) and recorded on 1 d and 9 d. Dead individuals of BHS were recorded and then removed from the box to avoid water contamination.

### 2.4. Determination of Protein, Glutamic Oxaloacetic Transaminase, Glutamic Pyruvic Transaminase, and Malondialdehyde in Black Helmet Snails

BHS were frozen to death in a −20 °C freezer. The snail tissues were weighed before adding physiological saline at a ratio of 1:9 (*w*:*v*). Foot and hepatopancreas of BHS were carefully collected using stainless sieve according to published anatomy structure [29]. The foot tissue was used to determine the protein content. The tissues were homogenized in the solution in an ice bath and then centrifuged at 2500 r/min for 10 min. The supernatant obtained is used to determine enzyme and protein content. The activities of glutamic oxaloacetic transaminase (GOT, μmol/g) and glutamic pyruvic transaminase (GPT, μmol/g) in the tissue of GAS were determined accordingly. Enzyme extract was added to a substrate solution (pH 7.4) containing the 2 mM alpha oxoglutamic acid and 200 mM DL aspartic acid (GOT) or 200 mM DL alanine (GPT). The mixture was incubated at 37 °C for 1 h, and the reaction was terminated by adding 2,4-dinitrophenylhydrazine. After incubating at 37 °C for 20 min, the mixture was added with 5 mL of 0.4 M NaOH. The resultant solution was measured at 500 nm. The protein content (mg/g) was determined using Coomassie Brilliant Blue [30]. The malondialdehyde (MDA, μmol/g) content of the snail was determined using thiobarbituric acid with the malondialdehyde detection kit (AKFA013C, Beijing Box Technology Co., Ltd., Beijing, China) [31].

### 2.5. Observation of the Hepatopancreas Structure of BHS

The stained sections of the tissue of BHS at 48 h were observed through Hematoxylin Eosin staining method [32]. The hepatopancreas tissue of the BHS was fixed with 4% paraformaldehyde, embedded with paraffin, solidified with cold before obtaining the sliced tissue. The nucleus and cytoplasm were stained separately with hematoxylin and eosin staining solution. After staining, the film was sealed with neutral resin and observed, and images were collected using a Nikon optical microscope (Nikon Eclipse E100, Nikon, Tokyo, Japan).

### 2.6. Statistics and Analyses

Experimental data were sorted using Excel 2016 (Microsoft, Washington, DC, USA). Normality tests, homogeneity of variance tests, and one-way analysis of variance were conducted through SPSS (version 26.0, IBM, Armonk, NY, USA). Multiple comparisons were conducted to test significant differences through Tukey’s HSD (*p* < 0.05 or 0.01). The LD50 of the contaminated water against BHS was calculated using the Bliss method. Origin software (version 2021, OriginLab, Northampton, MA, USA) was used to obtain figures.

## 3. Results

### 3.1. Changes in pH of the Contaminated Water

**Figure 1:**
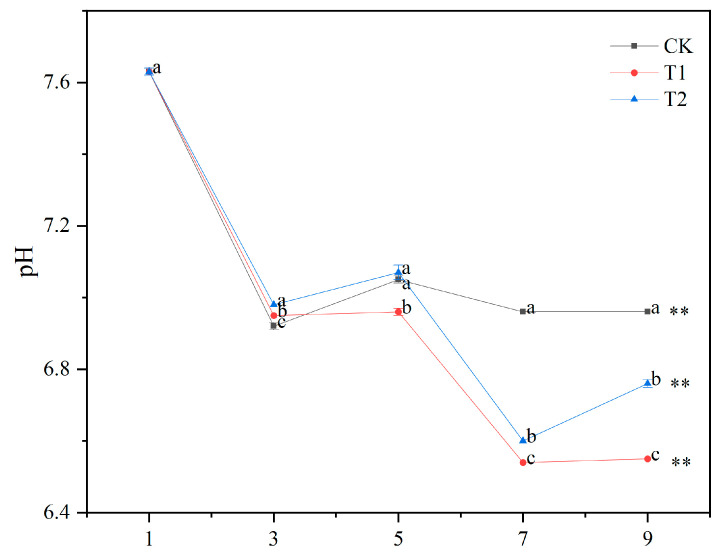
Changes in pH of water contaminated by metabolic activity of golden apple snail (GAS, *Pomacea canaliculata*) under different feeding conditions. Note: CK—no feeding; T1—water contaminated by metabolic activity of GAS feeding on *Acanthus ilicifolius*; T2—water contaminated by metabolic activity of GAS feeding on *Sonneratia apetala*. Values with different lowercase letters are significantly different between T1, T2 and CK at *p* < 0.01 or 0.05. Two asterisks indicated a significant difference in values of T1, T2 and CK between 9 d and 1 d at *p* < 0.01.

The pH values of the contaminated water in T1, T2, and CK changed significantly after 3 days (Figure 1). The pH values of the contaminated water of T1, T2, and CK showed an overall decreasing trend, ranging from 6.5 to 7.7. The pH of the contaminated water from 7 to 9 days was in the order T1 < T2 < CK (*p* < 0.05). At 9 d, the pH values of the contaminated water in T1, T2, and CK decreased by 14%, 11%, and 9% from the initial values, respectively. At 9 d, the pH values of the contaminated water in T1 and T2 were significantly lower than the CK by 6% and 3%.

### 3.2. Changes in DO and COD of the Contaminated Water

**Figure 2:**
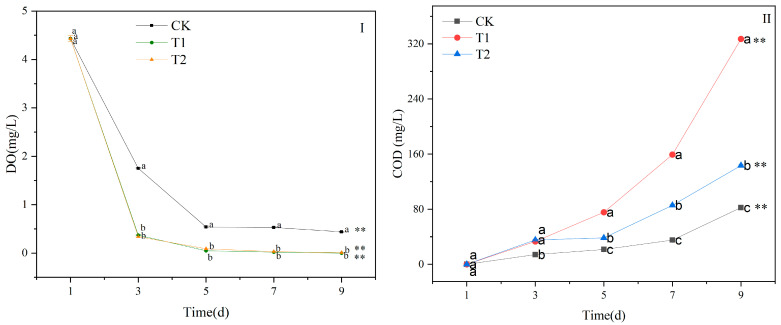
Changes in dissolved oxygen (DO, (**I**)) and chemical oxygen demand (COD, (**II**)) of water contaminated by metabolic activity of golden apple snail (GAS, *Pomacea canaliculata*) in T1, T2 and CK. Note: CK—no feeding; T1—water contaminated by metabolic activity of GAS feeding on *Acanthus ilicifolius*; T2—water contaminated by metabolic activity of GAS feeding on *Sonneratia apetala*. Values with different lowercase letters are significantly different between T1, T2 and CK at *p* < 0.01 or 0.05. Two asterisks indicated a significant difference in values of T1, T2 and CK between 9 d and 1 d at *p* < 0.01.

The dissolved oxygen (DO) and COD of the contaminated water in T1, T2, and CK changed significantly after 1 d (Figure 2). The DO content of T1, T2, and CK continuously decreased from 1 d to 9 d. The DO content in the T1 and T2 treatments was significantly lower than that in CK after 1 d (*p* < 0.05), and no difference was observed between T1 and T2. At 9 d, the DO content in the T1 and T2 treatments approached zero (Figure 2I). The COD content of T1, T2, and CK increased steadily with the incubation time (Figure 2II). At 3 d, the COD content of the T1 and T2 treatments was significantly higher than CK (*p* < 0.05). From 5 d to 9 d, the COD content of the T1 treatment was significantly higher than that of the T2 treatment (*p* < 0.05). At 9 d, the COD content of the T1 and T2 treatments increased by 297% and 74%, respectively, compared to CK (*p* < 0.05).

### 3.3. Changes in Contents of N and P in the Contaminated Water

**Figure 3:**
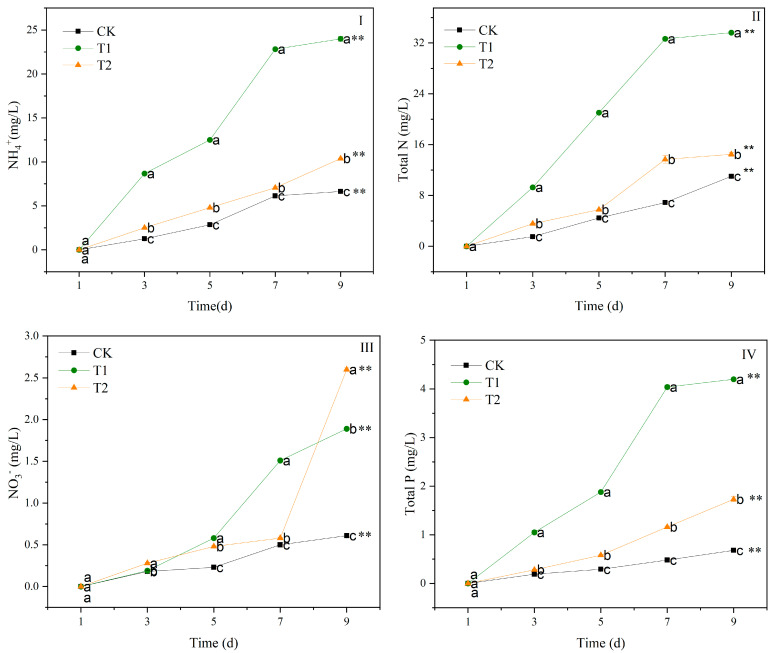
Changes in NH_4_^+^ (**I**), TN (**II**), NO_3_^−^ (**III**), and TP (**IV**) of water contaminated by metabolic activity of of golden apple snail (GAS, *Pomacea canaliculata*) in T1, T2 and CK. Note: CK—no feeding; T1—water contaminated by metabolic activity of GAS feeding on *Acanthus ilicifolius*; T2—water contaminated by metabolic activity of GAS feeding on *Sonneratia apetala*. Values with different lowercase letters are significantly different between T1, T2 and CK at *p* < 0.01 or 0.05. Two asterisks indicated a significant difference in values of T1, T2 and CK between 9 d and 1 d at *p* < 0.01.

The quality indicators, including ammonium (NH_4_^+^), total nitrogen (TN), nitrate (NO_3_^−^), and total phosphorus (TP) of the contaminated water, changed significantly in T1 and T2 after 9 days (*p* < 0.01) (Figure 3). The NH_4_^+^, TN, NO_3_^−^, and TP contents of the contaminated water in T1 and T2 treatments increased with the incubation time. From 3 d to 9 d, the NH_4_^+^ content was T1 > T2 > CK (*p* < 0.05) (Figure 3II). At 9 d, the NH_4_^+^ content in the contaminated water in T1 and T2 increased by 262% and 57% compared to CK. The changes in the TN content are similar to those of the NH_4_^+^ of the contaminated water in T1 and T2 (Figure 3II). The TN content of the contaminated water from 3 d to 9 d was T1 > T2 > CK (*p* < 0.05). The TN content of the contaminated water in the T1 and T2 treatments increased by 205% and 31% compared to CK at 9 d (*p* < 0.05). The T1 and T2 treatments significantly increased the NO_3_^−^ content in the contaminated water (Figure 3III). At 9 d, the NO_3_^−^ content in the T1 and T2 treatments increased by 210% and 326% compared to the CK (*p* < 0.05). After 1 d, the TP content of the contaminated water in the T1 and T2 treatments was always higher than that in CK, decreasing in the order T1 > T2 > CK (*p* < 0.05) (Figure 3IV). At 9 d, the TP content in the T1 and T2 treatments increased by 518% and 154% compared to CK (*p* < 0.05).

### 3.4. Changes in the Mortality of Black Helmet Snails

**Figure 4:**
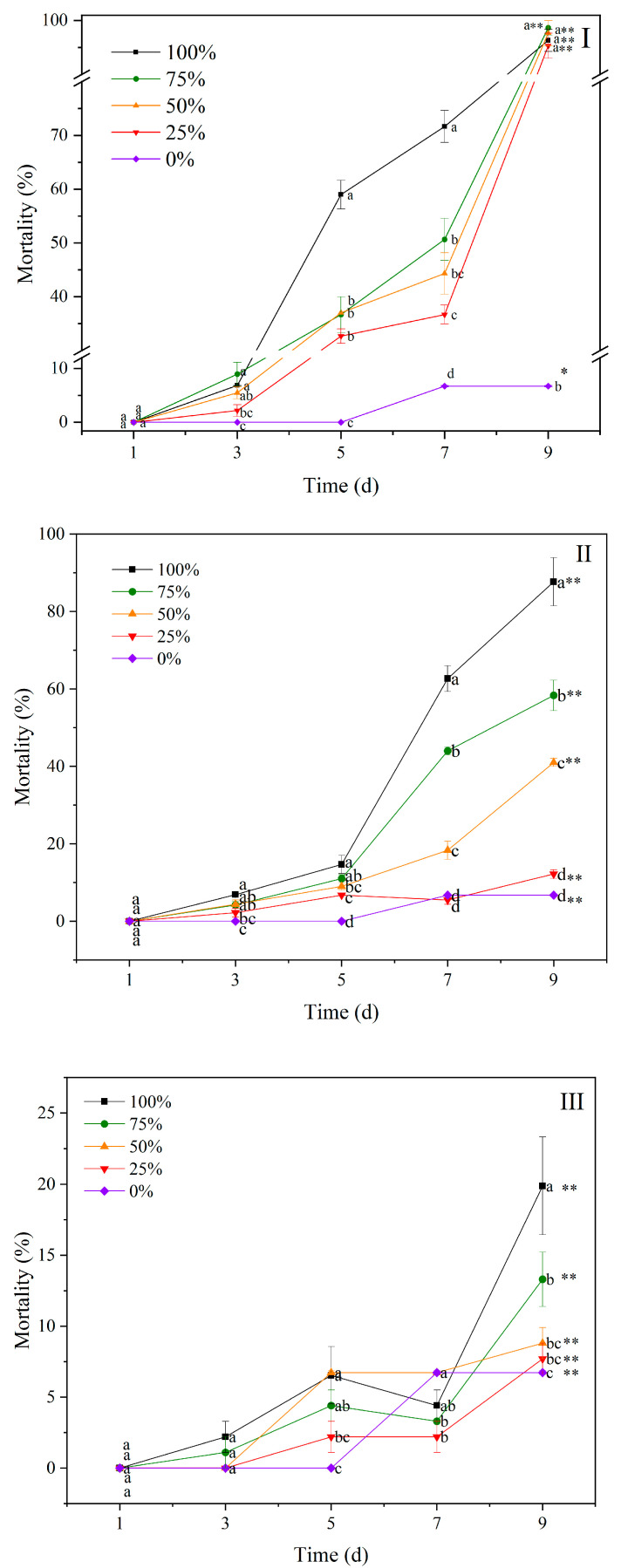
Mortality of black helmet snails exposed to water contaminated by metabolic activity of golden apple snail (GAS, *Pomacea canaliculata*) in water contaminated by metabolic activity of GAS feeding on *Acanthus ilicifolius* (**I**), water contaminated by metabolic activity of GAS feeding on *Sonneratia apetala* (**II**), and no feeding (**III**). Note: For 100% of the original solution of the contaminated water; 75%—diluted to 75% of the original solution; 50%—diluted to 50% of the original solution; 25%—diluted to 25% of the original solution; 0—2.5‰ saline solution. Values with different lowercase letters are significantly different at *p* < 0.01 or 0.05. One or two asterisks indicated a significant difference in values of five gradients between 9 d and 1 d at *p* < 0.05 or 0.01.

The contaminated water led to differential mortalities and weights of the BHS (Figure 4). At 9 d, the mortality of the BHS exposed to the 100% contaminated water from T1 was 96% (Figure 4I), followed by those from T2 (87%) (Figure 4II), and the mortality of the BHS exposed to the contaminated water in CK was less than 20% (Figure 4III). There was a significant difference in the mortality of the BHS between the five dilution gradients of the contaminated water in T1, T2, and CK at 9 d (*p* < 0.05), indicating the GAS’s metabolic substance negatively affects the survival of the BHS. At 9 d, the mortality of BHS exposed to the contaminated water in T1, with the concentration ranging from 25% to 100%, was significantly higher than that of 0%. The mortality of BHS exposed to the contaminated water in T2 increased with the increase in concentrations at 9 d. The LC50 of the contaminated water in the T1 and T2 treatments against BHS was 22.72% and 55.50% at 9 d.

There was a significant drop in the weight of the BHS exposed to the contaminated water in T1, with concentrations ranging from 25% to 100% after 9 days compared to those at 1 d (Supplementary Figure S1I). Compared to the 0% contaminated water in T1, the weight of BHS also significantly decreased when exposed to the contaminated water, with concentrations ranging from 25% to 100%. The weight of the BHS exposed to the 100% contaminated water in T2 significantly reduced compared to those at 1 d (Supplementary Figure S1II). At 9 d, weights of the BHS significantly decreased in the 100% contaminated water in T2 compared to the other concentrations. There was no change in the weights of the BHS exposed to CK in five concentrations (0% to 100%) after 9 days. The BHS showed a similar weight to those at 1 d (Supplementary Figure S1III).

### 3.5. Effect of the Contaminated Water on Protein, GOT, GPT, and MDA Contents

**Figure 5:**
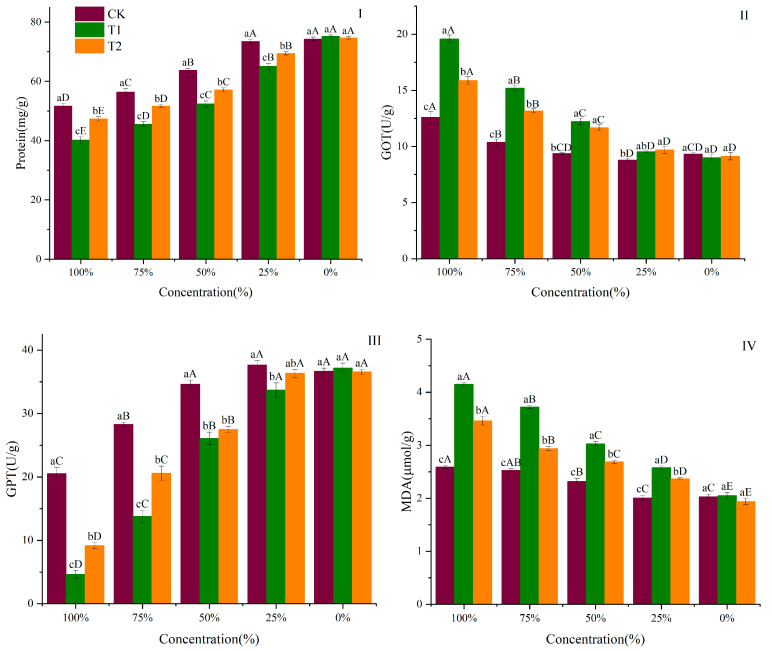
Protein (**I**), GOT (**II**), GPT (**III**), and MDA (**IV**) content of black helmet snails (BHS) exposed to different gradients of T1, T2, and CK. Note: CK—no feeding; T1—water contaminated by metabolic activity of GAS feeding on *Acanthus ilicifolius*; T2—water contaminated by metabolic activity of GAS feeding on *Sonneratia apetala*. For 100% the original solution of the contaminated water; 75%—diluted to 75% of the original solution; 50%—diluted to 50% of the original solution; 25%—diluted to 25% of the original solution; 0—2.5‰ saline solution. Foot was used to determine protein content. Hepatopancreas was used to determine GOT, GPT, and MDA content. Values with different lowercase letters are significantly different between CK, T1, and T2 at the same concentration at *p* < 0.01 or 0.05. Values with different uppercase letters are significantly different between five gradients at *p* < 0.01 or 0.05.

The protein, GOT, GPT, and MDA contents of the BHS significantly changed after exposure to different concentrations of the contaminated water in T1, T2, and CK (Figure 5). The BHS’s protein contents increased with the decreased concentration of the contaminated water of T1, T2, and CK (Figure 5I). The BHS exposed to the 100% contaminated water showed the lowest protein value. The protein content of the BHS was CK > T2 > T1 in the 100%, 75%, 50%, and 25% contaminated water (*p* < 0.05). The protein contents of the BHS exposed to the 100% contaminated water of CK, T1, and T2 treatments were 51.63 mg/g, 40.20 mg/g, and 47.33 mg/g, respectively.

The GOT content of the BHS decreased with the decrease in the concentrations of the contaminated water (Figure 5II). When exposed to the 100% and 75% contaminated water, the GOT contents of the BHS were T1 > T2 > CK (*p* < 0.05). The GOT content of the BHS exposed to the 100% contaminated water of T1 and T2 increased by 55% and 26%. A significant difference was observed in GOT contents between CK and T1 when BHS was exposed to the 50% contaminated water, and the GOT increased by 31% (*p* < 0.05).

The GPT contents of the BHS increased with the decrease in the concentrations of the contaminated water (Figure 5III). When exposed to the 100% and 75% contaminated water of T1, T2, and CK, the GPT contents of the BHS were CK > T2 > T1 (*p* < 0.05). There was no significant difference in GPT content between the T1 and T2 treatments at the 50% contaminated water. The GPT content of the BHS exposed to the 25% contaminated water in T1 decreased by 11% than that in CK (*p* < 0.05).

The MDA contents showed a significant decreasing trend with a decrease in the concentration of the contaminated water in T1 and T2 (Figure 5IV). Except for the 0% contaminated water, the MDA content of the BHS exposed to the contaminated water at each concentration was T1 > T2 > CK (*p* < 0.05). The content of MDA of the BHS exposed to the 100% contaminated water in T1 and T2 increased by 38% and 34% compared to CK. The MDA contents of the BHS exposed to the 100% contaminated water in T1 and T2 reached maximum values of 4.15 μmol/g and 3.46 μmol/g, respectively.

### 3.6. Changes in the Hepatopancreas Structure of BHS Exposed to the Contaminated Water

**Figure 6:**
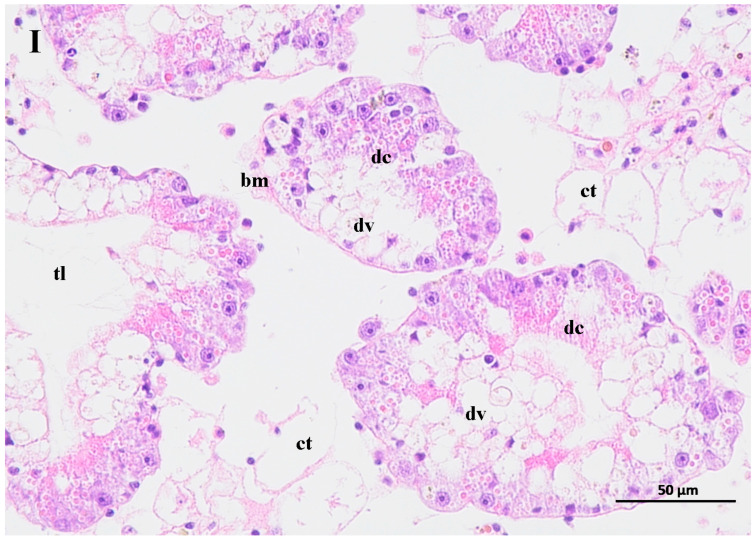

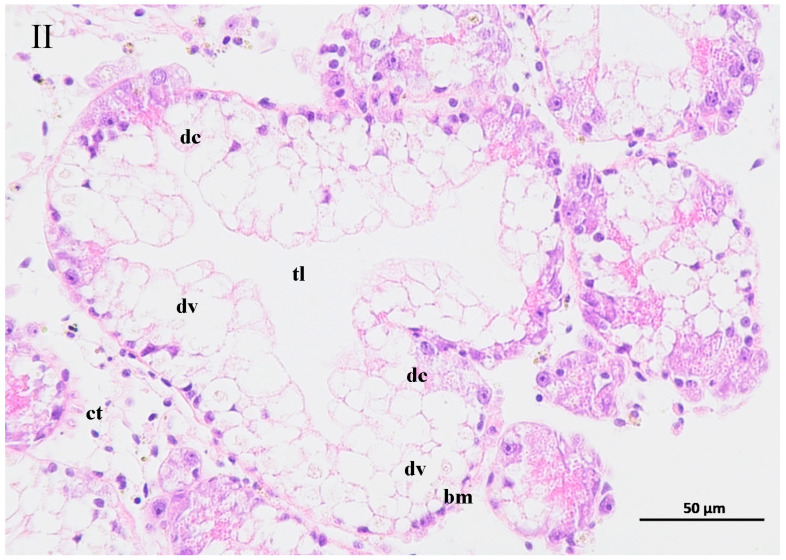

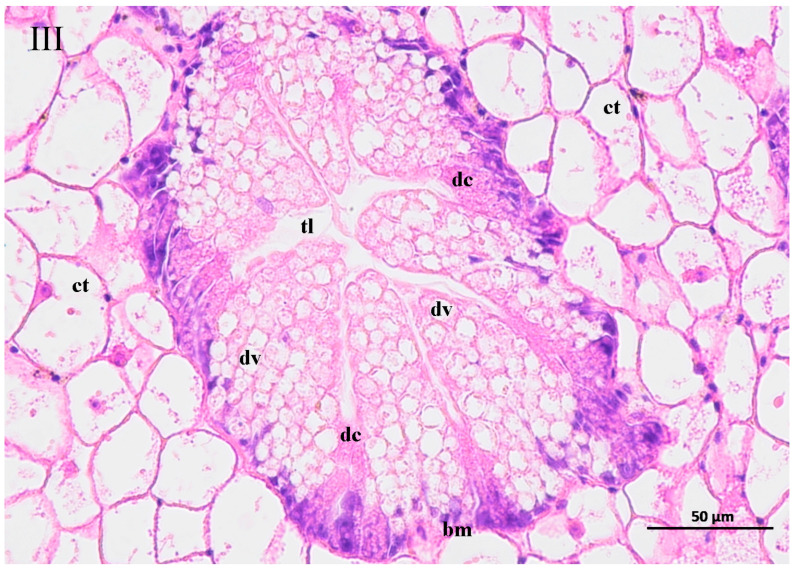
Change of hepatopancreas structure of black helmet snails exposed to water contaminated by metabolic activity of golden apple snail (GAS, *Pomacea canaliculata*). note: (**I**)—25% water contaminated by metabolic activity of GAS fed *Acanthus ilicifolius* (T1); (**II**)—50% water contaminated by metabolic activity of GAS fed *Sonneratia apetala* (T2); (**III**)—100% water contaminated by metabolic activity of GAS without feeding (CK). dc: digestive cell; dv: digestive vacuole; bm: basal membrane; ct: connective tissue; tl: tubule lumen.

The contaminated water of the GAS feeding on two mangrove leaves changed the structure of the hepatopancreas of the BHS (Figure 6). The hepatopancreas structure was disordered for the BHS exposed to the 25% contaminated water in the T1 treatment and the 50% contaminated water in the T2 treatment. An increase in the width of the tubule lumen was observed for the BHS in the T1 and T2 treatments (Figure 6I, II). The digestive cells of the BHS in T1 and T2 were degenerated. Incomplete digestive vacuoles were observed in the hepatopancreas of the BHS after exposure. The connective tissue lost its shape, and the basal membrane became thin or lost in the T1 and T2 treatments. For the BHS exposed to the 100% contaminated water in CK, there was a complete hepatopancreas structure, and only some cell degeneration was observed in the tissue after exposure (Figure 6III).

## 4. Discussion

### 4.1. Effects of Feeding Two Mangroves on the Water Quality

The GAS has a wide range of dietary habits, not only feeding on mangroves such as *Acanthus ilicifolius*, *Sonneratia apetala*, *Kandelia candel*, and *Aegiceras corniculatum* [18] but also directly feeding on macrophytes and preying on small aquatic animals in a freshwater habitat [33,34].

The pH, DO, and COD are critical environmental factors that can regulate the physicochemical properties and affect the growth, development, reproduction, and survival of aquatic organisms. The excrement of GAS contains a high amount of uric acid, which leads to water acidification [35]. Meanwhile, the GAS activity also releases carbon dioxide, leading to a decrease in water pH value. The metabolic activity of GAS decreased the dissolved oxygen (DO) after feeding on *Acanthus ilicifolius* and *Sonneratia apetala*. The oxygen-consuming effect of this process resulted in significantly lower DO levels in the feeding treatment compared to the control without feeding. The accumulation of water-soluble organic matter released by the GAS in the contaminated water enhanced the decomposition activity of microorganisms, which also consumed a large amount of DO in the water.

The GAS has amphibious characteristics and a highly tolerant enzyme system, which makes it highly tolerant to low-oxygen water habitats [36]. In this study, the COD in the contaminated water of the GAS fed on *Acanthus ilicifolius* was higher than that of the *Sonneratia apetala* treatment, indicating the organic pollutant content in the contaminated water was also high. A previous study found that the GAS prefers feeding on *Acanthus ilicifolius* compared to *Sonneratia apetala* [18,19]. It is possible that feeding on *A. ilicifolius* leads to faster digestion and higher emission of metabolite waste. However, no enzymetic and digestive traits of GAS feeding on mangrove leaves were reported and further research was necessary to understand the phenomenon.

### 4.2. Changes in Nitrogen and Phosphorous Contents in the Contaminated Water

In this study, the different nitrogen forms in the contaminated water, including ammonium, nitrite, and nitrate, released by GAS significantly increased. The metabolism of the GAS promoted nitrogen accumulation, which possibly led to the deterioration of water quality. The GAS prefers to feed on plants with a high nitrogen content [34], and they often release a large amount of excrement, with nitrogen mainly present in the form of uric acid or ammonium [37]. Compared to *Sonneratia apetala*, the nitrogen content in the leaves of *Acanthus ilicifolius* was higher, which explained the phenomenon that total nitrogen and ammonium in the contaminated water of *A. ilicifolius* treatment after digestion were significantly higher than those of *Sonneratia apetala* treatment.

Ammonium in water can be converted into nitrate under nitrification. In this study, the proportion of nitrate in the contaminated water was relatively low, indicating that nitrification function was weak. This may be related to the inhibition of the nitrification process by acidification of the contaminated water [38]. Nitrification is a process that consumes oxygen and ammonium (NH_4_^+^) and supplies nitrate (NO_3_^−^) [39]. In this study, the DO in the contaminated water decreased rapidly, and the NH_4_^+^ accumulation occurred in low-oxygen environments.

The inorganic N and P contents in the contaminated water far exceeded the eutrophication standard of water. The GAS can survive in severely eutrophic water with the P content reaching 1 mg/L [5]. There was a significant difference in the total P content in the contaminated water due to the feeding preference of GAS. GAS had a higher preference for feeding on *Acanthus ilicifolius* than *Sonneratia apetala* [18], which is consistent with the variation pattern of COD in the contaminated water. We speculated that the GAS feeding on *Acanthus ilicifolius* excreted much P in their metabolic waste, which may be related to the difference in P content of the two mangroves.

### 4.3. Toxicity of the Contaminated Water Against BHS

In this study, the NH_4_^+^ content in the contaminated water significantly increased, which caused significant toxicity to black helmet snails. On 9 d, the contaminated water of GAS feeding on *Acanthus ilicifolius* resulted in a 100% mortality of black helmet snails. The NH_4_^+^ in water affects aquatic animals’ behavior, growth, respiration, immune system, and antioxidant system, and a high concentration of NH_4_^+^ can lead to acute toxicity in organisms [3,40]. The NH_4_^+^ interferes with mitochondrial function and produces excess reactive oxygen species, which causes peroxidation to produce MDA and leads to loss of cell function and dysfunction of the antioxidant system in largemouth bass (*Micropterus salmoides*) [41]. As an indicator for evaluating hepatopancreas function, GOT is related to the level of the immune system of invertebrates [42]. The NH_4_^+^ toxicity led to an increase in the GOT activities of *ussuri cisco* (*Coregonus ussuriensis*) [43].

The LC50 of the contaminated water of the GAS feeding on *Acanthus ilicifolius* on black helmet snails within 9 days reached 22.72%, indicating a higher toxicity than that of GAS feeding on *Sonneratia apetala*. Mangrove leaves usually contain more secondary metabolites with a growth inhibitory activity [44]. In addition to the toxicity caused by basic properties and nitrogen and phosphorus content, there may be other secondary metabolite toxicity in the two contaminated waters. Aquatic organisms have different adaptability to water quality. The accumulation of metabolites from the GAS leads to the deterioration of water quality, which can cause the disappearance of some native species in the micro-habitat of mangroves. The high tolerance of the GAS allows them to gain more space and resources under such a scenario, which may be a mechanism assisting the successful invasion of the GAS in small-scale aquatic habitats in mangrove wetlands.

### 4.4. Effect of Contaminated Water on Physiological Traits of Native Snail

The hepatopancreas is an essential organ for the detoxification of gastropods under stress [45]. GOT and GPT activity are often used as indicators for evaluating tissue damage, as the GOT enzyme is positively correlated with the severity of cell damage [46]. In this study, the GOT enzyme activity of the black helmet snail increased with an increase in two contaminated water concentrations. Glutamic pyruvic transaminase (GPT) and glutamicoxalacetic transaminase (GOT) participate in the process of conversion of glutamate into a-ketoglutarate, which subsequently enters the TCA cycle as precursor metabolites [47]. The changes in GPT and GOT indicated that the GAS metabolites affected the protein and energy metabolism of the black helmet snail, causing damage to its normal function.

MDA is a metabolic product of membrane unsaturated fatty acid peroxidation caused by oxygen free radicals, reflecting oxidative damage to organisms [48]. In this study, the increase in the contaminated water concentration caused high levels of MDA content in the tissue of black helmet snails fed on *Acanthus ilicifolius*, and the lipid peroxidation produced by black helmet snails also increased. The black helmet snail in the contaminated water of GAS feeding on *Acanthus ilicifolius* was subjected to higher stress than that of *Sonneratia apetala*, and the degree of cell damage of the black helmet snail was also higher. The phenomenon was confirmed in the microscopy sections of the black helmet snail. The metabolic substances in water contaminated by GAS can cause extensive degeneration and even necrosis of tissue cells of the native snail, ultimately leading to high mortality of black helmet snails.

### 4.5. The Competitive Effect of GAS in Mangrove Wetlands

In the invaded area, the GAS may alter the water quality of local regions of mangrove wetlands, affecting the survival of the native BHS. However, this negative effect is related to the density of the GAS and the scale of the local habitat in mangrove wetlands. As an invasive animal, the GAS is prone to high-density aggregation when food is abundant. Under such a scenario, the metabolism of high-density GAS populations can cause changes in the water quality of local habitats in mangrove wetlands to some extent. For native snails, their density and metabolic activity are limited, resulting in a relatively low risk of water quality deterioration.

Meanwhile, GAS faces multiple natural enemies in its native area (South America), limiting its population expansion. It is a low risk for local snails in native areas to be affected by water quality changes of the GAS. However, there is limited research on the impact of GAS on local snails in their native area. In the invaded area, the metabolism of the GAS negatively affects the survival of native snails, which may be a pathway for invasion. However, this requires in situ monitoring to confirm the correlation between water quality and changes in native snail population. Overall, our results on the impact of the water quality by the GAS and the related disturbance on the BHS may be more applicable for understanding the invasion of high-density GAS populations in a local-scale mangrove habitat.

## 5. Conclusions

Alien aquatic animals change the physicochemical properties of the invaded habitats during colonization, showing a significant complex impact on native communities. This study analyzed the characteristics of water contaminated by invasive golden apple snails under salt stress. Even under salinity stress, we found that the GAS metabolic activity led to water deterioration. The GAS decreased the water pH and consumed a large amount of DO in the water, leading to increases in COD, total P, total N, NH_4_^+^, and NO_3_^−^. As a result, the contaminated water of the GAS was toxic to native mangrove black helmet snails. The hepatopancreas tissue of the GAS was significantly damaged, and the contaminated water of the GAS that fed on *Acanthus ilicifolius* inhibited the survival ratio and physiological traits of the local black helmet snails. This phenomenon indicates that during the GAS invasion into the local aquatic habitats in mangrove wetlands, its population colonization was accompanied by an indirect competitive effect on the native snail. The invasion of the GAS into mangrove wetlands can alter benthic biodiversity, and this invasion process is closely related to the mangrove’s species. For the mangrove wetlands of *Acanthus ilicifolius* and *Sonneratia apetala*, the native benthic gastropod biodiversity should be monitored regularly to avoid possible GAS invasion, and the risk assessment of GAS invasion should be carried out in these two mangroves.

## Figures and Tables

**Table 1:** Quality determination of metabolic solution.

Indicators	Measurement	Instrument	Citation
Temperature (°C)	-	Thermometer (Haiyi, Hengshui, China)	-
Dissolved oxygen (mg/L)	Coating electrode method	Dissolved oxygen analyzer (AR8010, SMART SENSOR, Dongguan, China);	[23]
Chemical oxygen demand (COD, mg/L)	Potassium dichromate method	Multi-parameter water quality detector (XZ-0125, Shanghai Haizheng Electronic Technology Co., Ltd., Shanghai, China)	[24]
pH	Electrode method	pH meter (SX713, LABSEN, Shanghai, China)	-
Ammonium (NH_4_^+^, mg/L)	Nessler reagent Spectrophotometry	Multi-parameter water quality detector (XZ-0125, Shanghai Haizheng Electronic Technology Co., Ltd., Shanghai, China)	[25]
Nitrate (NO_3_^−^, mg/L)	Ultraviolet Spectrophotometry	UV spectrophotometer (UV-1600PC, Juneng Instrument Equipment Co., Ltd., Jiaxing, China)	[26]
Total phosphorus (TP, mg/L)	Ammonium molybdate Spectrophotometry	UV spectrophotometer (UV-1600PC, Juneng Instrument Equipment Co., Ltd., Jiaxing, China)	[27]
Total nitrogen (TN, mg/L)	Ultraviolet Spectrophotometry of Basic potassium persulfate digestion	UV spectrophotometer (UV-1600PC, Juneng Instrument Equipment Co., Ltd., Jiaxing, China)	[28]

## Data Availability

https://figshare.com/s/e12d0c2f6ca14228324a accessed on 6 November 2024.

## Supplementary Materials

The following supporting information can be downloaded at: www.mdpi.com/article/10.3390/biology14020141/s1, Supplementary Figure S1 Weight of black helmet snails (BHS) exposed to the contaminated water in T1(I), T2(II), and CK(III).
