# Supplementary material for: Metabolic Activity of Invasive Apple Snails Negatively Affects the Survival of Native Benthic Snail in Mangrove
Source: Biology (Basel). 2025 Jan 29;14(2):141. doi: 10.3390/biology14020141 (PMC11851716; doi:10.3390/biology14020141)
Supplement: Supplementary file 1 [file biology-14-00141-s001.zip › biology-3328681-supplementary.pdf]

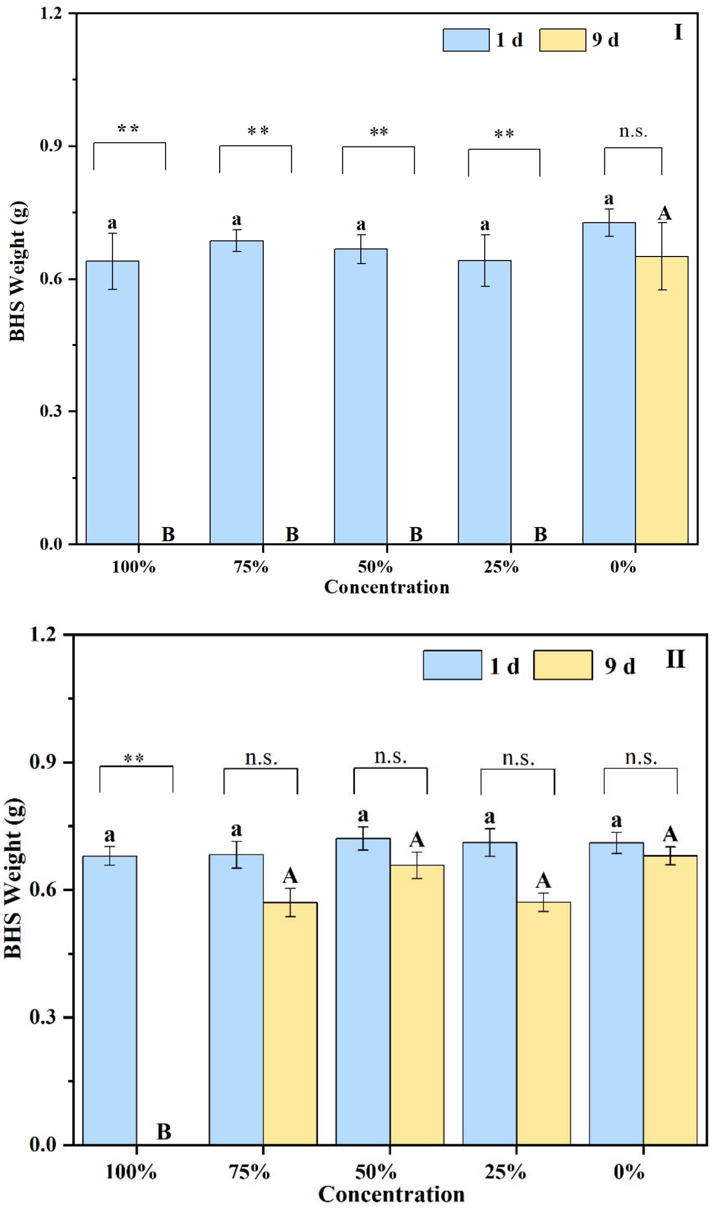

I
1 d
9 d
BHS Weight (g)
1.2
0.9
0.6
0.3
0.0
**
**
**
**
n.s.
a
a
a
a
a
A
B
B
B
B
100%
75%
50%
25%
0%
Concentration
II
1 d
9 d
BHS Weight (g)
1.2
0.9
0.6
0.3
0.0
**
n.s.
n.s.
n.s.
n.s.
a
a
a
a
a
A
A
A
A
B
100%
75%
50%
25%
0%
Concentration

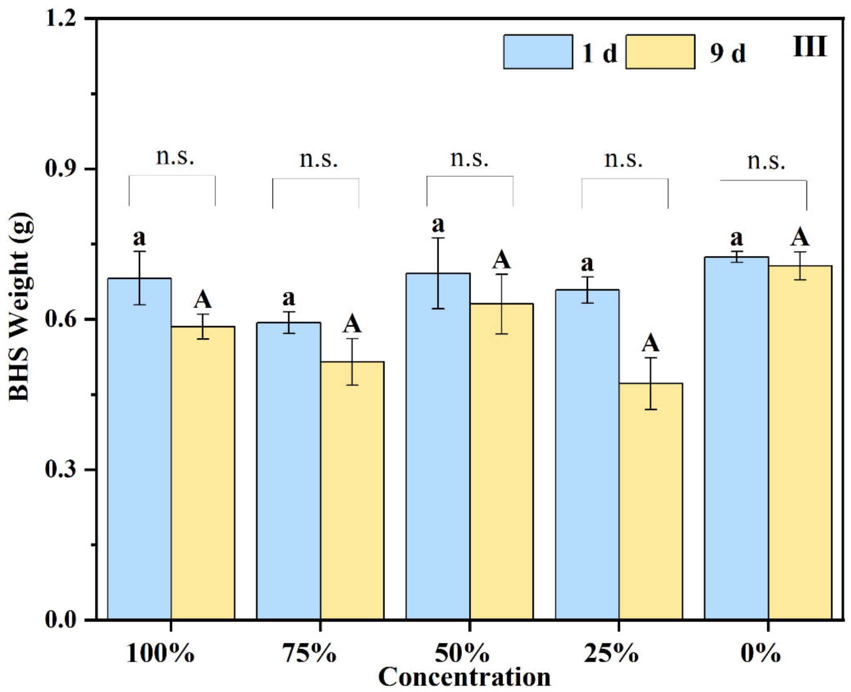

Supplementary Figure S1 Weight of black helmet snails (BHS) exposed to the contaminated water in T1(I), T2(II), and CK(III)

Note: CK—no feeding; T1—water contaminated by metabolic activity of GAS feeding on *Acanthus ilicifolius;* T2—water contaminated by metabolic activity of GAS feeding on *Sonneratia apetala*. 100%—the original solution of the metabolic water; 75%—diluted to 75% of the original solution; 50%—diluted to 50% of the original solution; 25%—diluted to 25% of the original solution; 0%—2.5‰ saline solution. Different lowercase and uppercase letters indicated values in four concentrations of T1, T2 and CK was significant at 1 d and 9 d at $p < 0.01$ or 0.05. The two asterisks indicated a significant different in values of T1, T2 and CK between 9 d and 1 d at $p < 0.01$.
